# Complex Factors in Hydrocephalus Development in Tuberous Sclerosis Complex: A Case Report of Subependymal Giant Cell Astrocytoma

**DOI:** 10.7759/cureus.65132

**Published:** 2024-07-22

**Authors:** Hajime Nakamura, Masaki Izumi, Yoshinori Omori, Shingo Numoto, Ayataka Fujimoto

**Affiliations:** 1 Neurosurgery, Seirei Hamamatsu General Hospital, Hamamatsu, JPN; 2 Center of Epilepsy and Functional Neurology, Seirei Hamamatsu General Hospital, Hamamatsu, JPN; 3 Pediatric Neurology, Seirei Hamamatsu General Hospital, Hamamatsu, JPN

**Keywords:** tuberous sclerosis complex (tsc), mammalian target of rapamycin (mtor) inhibitor, surgical removal, hydrocephalus, subependymal giant cell astrocytoma (sega)

## Abstract

Subependymal giant cell astrocytoma (SEGA) associated with tuberous sclerosis complex (TSC) occurs in 5-20% of TSC patients, with a subset developing hydrocephalus. We present a case of a 14-year-old male diagnosed with TSC in the neonatal period who developed SEGA and subsequent hydrocephalus. Despite reducing the tumor size with the mammalian target of rapamycin (mTOR) inhibitors, ventricular enlargement persisted, indicating that obstructive hydrocephalus due to the foramen of Monro blockage was not the sole mechanism. Elevated cerebrospinal fluid (CSF) protein levels suggested additional factors like impaired CSF outflow. This case underscores the need for comprehensive treatment strategies and further research to better understand and manage hydrocephalus in TSC patients with SEGA.

## Introduction

Subependymal giant cell astrocytoma (SEGA) associated with tuberous sclerosis complex (TSC) occurs in 5-20% of TSC patients [1]. Approximately 4-14% of these cases present with hydrocephalus, and ventricular dilatations are seen in 85% [2,3]. Studies have indicated that the mechanism by which SEGA causes hydrocephalus involves enlargement of the SEGA leading to blockage of the foramen of Monro and the subsequent development of hydrocephalus [3-5]. However, three case reports involving four patients have suggested that hyperosmolarity due to hyperproteinorrhachia causes non-obstructive hydrocephalus [6-8].

The risk of obstructive hydrocephalus increases when a SEGA near the foramen Monro exceeds 1 cm, and resection is desirable when the tumor reaches about 2-3 cm [1,9]. However, if the sole mechanism were that SEGA caused obstructive hydrocephalus by blocking the foramen of Monro, the relationship between tumor size and hydrocephalus would be relatively straightforward. In clinical practice, cases where acute hydrocephalus occurs despite regular imaging on follow-up have been observed. Likewise, cases where hydrocephalus does not arise even with tumor enlargement have also been described. A simple mechanism of obstructive hydrocephalus caused by SEGA blocking the foramen of Monro therefore cannot explain such scenarios.

In this report, we present a case of SEGA associated with TSC where multiple factors were likely involved in the development of hydrocephalus, indicating that the theory of SEGA blocking the foramen of Monro alone is insufficient. This case has been reported in accordance with the CARE Guidelines: Consensus-based Clinical Case Reporting Guideline Development [10].

## Case presentation

The patient was a 14-year-old male who had been diagnosed with TSC in the neonatal period. MRI of the head showed multiple cortical tubers bilaterally in the cerebral cortex and SEGA near the right foramen of Monro. The patient had begun experiencing epileptic seizures at 11 years old. Genetic testing had not been conducted, but no family members were known to have exhibited clinical symptoms suggestive of TSC. According to the Wechsler Intelligence Scale for Children-IV, the patient had a full intelligence quotient of 67 and was a junior high school student attending a special education class. In terms of medical history, cardiac rhabdomyoma had been identified in the neonatal period and had regressed over time. At the time of presentation, he was on two anti-seizure medications (perampanel and lacosamide).

**Figure 1:**
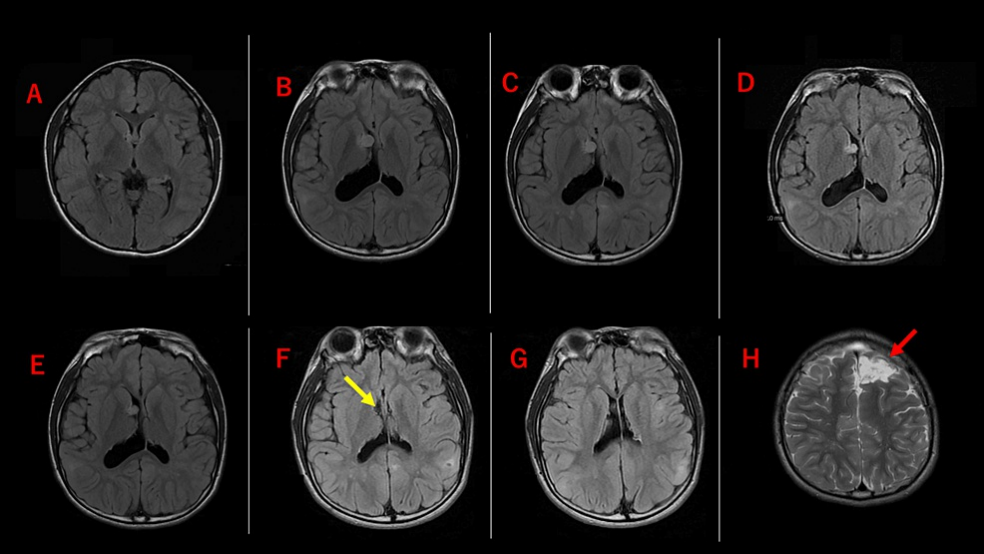
Changes in the size of the SEGA and ventricles over time Figures A, B, C, D, and E show preoperative head MRI, and Figures F, G, and H show postoperative head MRI. In June X-1, SEGA present near the foramen of Monro in the right lateral ventricle was small and no ventricular enlargement was identifiable (A). A follow-up MRI approximately one year later in May X showed that both the SEGA and bilateral ventricles had enlarged with a right-side predominance (B). There was a discussion about whether to remove the SEGA surgically or to manage it with mTOR inhibitors; however, since there was no increased intracranial pressure, we used mTOR inhibitors. No symptoms of increased intracranial pressure were noted at that time, and hence mTOR inhibitor was first used. After approximately five months of treatment with mTOR inhibitor, in October X, the SEGA had reduced in size, but the ventricle size remained basically unchanged (E). Also shown are MRIs that were taken one month (C) and three months (D) after starting the mTOR inhibitor. A transcallosal approach was therefore used to resect the SEGA. Resection of the left frontal lobe, identified as a possible epileptogenic zone in a subdural electrode study, was performed simultaneously (H). The SEGA was completely resected (F), and ventricle size normalized (G). The disappearance of epileptic seizures suggests that the epileptogenic zone was successfully resected MRI: magnetic resonance imaging; mTOR: mammalian target of rapamycin; SEGA: subependymal giant cell astrocytoma

At 12 years old, a serial MRI of the head revealed that the SEGA was enlarging, reaching approximately 12 mm in diameter, and enlarged bilateral lateral ventricles, more pronounced on the right side, were evident (Figure 1). There was a discussion about whether to remove the SEGA surgically or to manage it with a mammalian target of rapamycin (mTOR) inhibitor; however, since the patient had not exhibited any symptoms of increased intracranial pressure, he was first given everolimus at 4 mg/day (based on a body surface area of 1.25 m^2^) instead of surgical resection. The tumor subsequently decreased in size from 12 mm to 7.7 mm with mTOR inhibitor treatment, but ventricular enlargement remained unchanged. Surgical intervention for the tumor was therefore planned.

To address the epilepsy, focal resection was also planned. Subdural electrode placement was performed to allow for the resection of the predicted epileptic focus simultaneous with SEGA resection. The SEGA was completely removed via a transcallosal approach. The SEGA was soft and about half of the tumor could be removed with biopsy forceps, while the remainder was easily resected with an ultrasonic aspirator. The foramen of Monro was patent with no obstruction and no adhesion. The seizure onset area in the left frontal lobe indicated in the subdural electrode study was also resected.

Pathological diagnosis confirmed the lateral ventricular tumor as SEGA and the tubers displayed cortical dysplasia. When approaching the ventricle, cerebrospinal fluid (CSF) was first collected from the ventricle using a venous catheter through the ependyma to prevent the entry of blood and other substances. Intraoperative CSF showed elevated proteins, normal glucose, and a slightly elevated cell count (Table 1).

**Table 1:** Intraoperative CSF findings CSF: cerebrospinal fluid

Parameter	Results	Reference range
Protein	123 mg/dL	15-45 mg/dL
Glucose	70 mg/dL	50-75 mg/dL
Cell count	20/μL	<5 /μL
Mononuclear cells	15/μL	
Multinuclear cells	5/μL	

Three months after the surgery, the patient was found to have experienced no major complications. Ventricular enlargement improved and no recurrence of either the tumor or seizures has been seen as of one year of follow-up.

## Discussion

This case was unique in that, despite the SEGA being reduced in size to less than 1 cm with mTOR inhibitor, enlargement of the lateral ventricle remained. This finding suggests that the mechanism of SEGA associated with TSC causing obstructive hydrocephalus cannot be solely attributed to the physical blockage of the foramen of Monro by the SEGA. Prior studies have indicated that mTOR inhibitors can be effective in managing SEGA and associated hydrocephalus by reducing tumor size [5,11]. However, our case demonstrates that even with significant tumor shrinkage, ventricular enlargement can persist, indicating additional factors at play.

Hydrocephalus caused by SEGA can sometimes be managed based on the concept of obstructive hydrocephalus using mTOR inhibitors, as previously documented [12,13]. For example, impaired CSF outflow and elevated CSF levels of protein are considered the most likely causes of hydrocephalus in other benign brain tumors such as vestibular schwannoma, meningioma, and ependymoma [14-16]. In our case, the intraoperative level of CSF protein was as high as 123 mg/dL, which is consistent with findings from studies on hydrocephalus associated with other benign tumors. Although the nature of the tumors differs between vestibular schwannoma and SEGA, the similarity in elevated CSF protein levels suggests that the underlying mechanism of hydrocephalus may involve impaired CSF circulation or increased osmotic pressure within the ventricles, rather than just physical obstruction [2,17]. This case report supports the hypothesis that factors other than tumor size contribute to hydrocephalus in patients with SEGA and TSC.

While this report is limited to a single instance and hence its findings cannot be generalized, it highlights the potential limitations of relying solely on mTOR inhibitors for hydrocephalus management in SEGA cases. In addition, it is necessary to mention that the fact that intracranial electrodes were also placed, before the SEGA resection, means that we cannot completely rule out the possibility of increased protein levels in CSF affected by the electrode surgery. Future research should focus on elucidating the precise mechanisms of hydrocephalus in SEGA patients and exploring alternative or adjunctive treatments to mTOR inhibitors.

## Conclusions

This case report highlights the complexity of managing SEGA associated with TSC and the development of hydrocephalus. Despite the successful reduction of SEGA size with mTOR inhibitor therapy, ventricular enlargement persisted, suggesting that the obstruction of the foramen of Monro is not the sole mechanism of hydrocephalus in these cases. The elevated intraoperative CSF protein levels indicate that additional factors, such as impaired CSF outflow or increased osmotic pressure within the ventricles, may play a significant role. This case underscores the importance of a comprehensive approach in diagnosing and treating hydrocephalus associated with SEGA, beyond merely focusing on tumor size reduction. Further research is necessary to fully understand the underlying mechanisms and to optimize treatment strategies for patients with SEGA and TSC.
